# Gellan Gum-Based In Situ Hydrogels for Nasal Delivery of Polymeric Micelles Loaded with Risperidone

**DOI:** 10.3390/gels11060404

**Published:** 2025-05-28

**Authors:** Bence Sipos, Mária Budai-Szűcs, Gábor Katona, Ildikó Csóka

**Affiliations:** Institute of Pharmaceutical Technology and Regulatory Affairs, University of Szeged, Eötvös Street 6, H-6720 Szeged, Hungary; budai-szucs.maria@szte.hu (M.B.-S.); csoka.ildiko@szte.hu (I.C.)

**Keywords:** gellan gum, in situ gel, hydrogel, cellulose derivative, nasal delivery, polymeric micelle

## Abstract

Nasal drug delivery faces numerous challenges related to the ineffectiveness of most nasal formulations without a mucoadhesive nature, prolonging residence time on the nasal mucosa. Another challenge is the low administrable dosage strength, which can be solved via nano-encapsulation techniques, including the utilization of polymeric micelles. In this study, gellan gum–cellulose derivative complex in situ gelling matrices were formulated to test their effect on the colloidal characteristics of polymeric micelles, their respective rheological behavior, and nasal applicability. It has been proven that these complex matrices can form gels upon contact with nasal fluid without disrupting the micellar structure. Changes in the drug release and permeation profile have been shown in a concentration-dependent manner to hinder the burst-like drug release profile of polymeric micelles. Formulations show concentration- and composition-dependent mucoadhesive features under nasal conditions. Most of the hydrogels possess a soft gel characteristic, making them suitable for nasal administration. In conclusion, this descriptive study provides useful insights for conscious, nasal dosage form design.

## 1. Introduction

Drug delivery through the nasal cavity plays a crucial role in current research and development trends due to its auspicious nature. The intranasal administration role has multiple advantages that can be exploited for most small-molecular-weight drugs, but even for peptides or antigens, it is a promising alternative to conventional administration routes [1,2]. The large and highly vascularized nasal mucosa offers great permeability to the blood vessels, making the nose-to-blood pathways feasible for systemic drug administration. Besides this pathway, direct nose-to-brain delivery can be achieved via the dense innervation of the nasal cavity by the trigeminal and olfactory nerves [3,4,5]. These nerves allow direct axonal transport for many drugs and nanocarriers, bypassing the blood–brain barrier, a crucial hindrance for many active substances [6,7].

Despite the advantageous nature, there are limitations and challenges regarding the nasal route. The average administrable volume ranges between 50 and 150 µL/nostril, limiting the administered drug concentration to the minimum per administration. This poses the technological requirement that the active substance should be highly concentrated, which can only be solved via solubilization techniques [8,9,10]. The other main challenge lies in the rapid elimination from the surface of the nasal mucosa due to the high rate of mucociliary clearance. To face these challenges, the combination of polymeric nanoparticles and in situ gelling hydrogels could be a suitable choice [11,12,13].

Polymeric micelles are built up by amphiphilic graft copolymers, capable of self-assembly above the critical micellar concentration and temperature. The main advantages of these polymers include the higher stability and solubilization degree compared to classic surfactants [3,14,15]. They are generally biodegradable and biocompatible, and the utilization of non-ionic polymers is less prone to irritation. Their average particle size ranges between 10 and 200 nm, making them suitable candidates for enhanced drug release and permeation through various biological barriers. Risperidone, the model drug applied during this study, has previously shown excellent nasal applicability for brain targeting. In vivo studies demonstrate that the encapsulation of risperidone in solid lipid nanoparticles showed a 10-fold higher brain concentration compared to its intravenous form [16]. Spanlastics were also studied in vivo, with higher drug targeting efficiency and direct transport percentage compared to their conventional counterparts [17]. The release and permeation kinetics of polymeric micelles are highly influenced by various factors, including pH, temperature, and whether there is an outer matrix in the formulation modifying the release profile [18,19,20,21].

**Figure 1:**
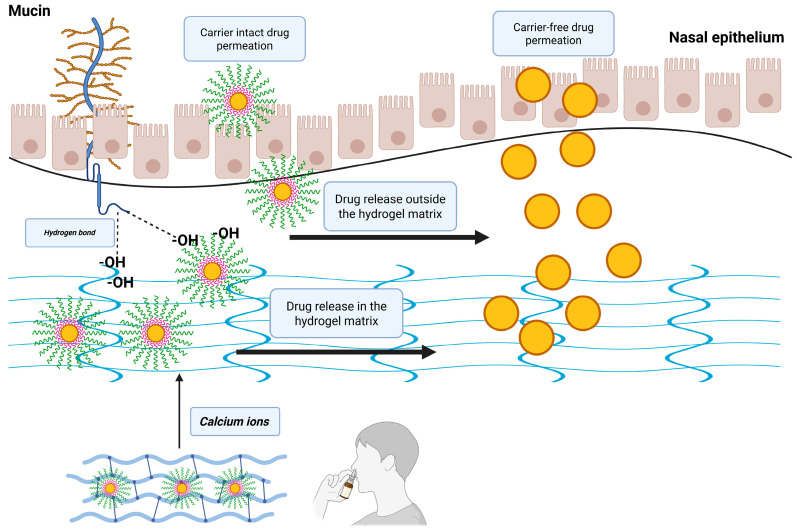
Schematic representation of the possible nasal administration of gellan gum–cellulose derivative matrices loaded with risperidone-encapsulated polymeric micelles.

One of the representatives of in situ gelling agents is gellan gum (GG), a water-soluble anionic polysaccharide produced by a bacterium, namely *Sphingomonas elodea*. It is a linear polysaccharide, composed of repeating tetrasaccharide units in the following structure: (→3)-β-D-glucose-(1→4)-β-D-glucoronic acid-(1→4)-β-D-glucose-(1→4)-α-L-rhammose-(1→) [22,23,24]. It is capable of gel formation upon contact with divalent (Ca^2+^, Mg^2+^) or monovalent (Na^+^, K^+^) ions, forming hydrogels. At ambient temperatures, without the presence of the mentioned ions, it is in a sol state, and by being administered to the nasal cavity, it can form hydrogels capable of increasing residence time [25,26,27]. The in situ gelling properties are influenced by the concentration of the ions and the addition of other polymers. Semisynthetic polymers are currently widely used in nasal delivery, including many cellulose derivatives, such as hydroxyethyl cellulose (HEC), methylcellulose (MC), and hydroxypropyl-methylcellulose (HPMC) [28,29,30,31,32]. A schematic representation of the administration and possible drug release and absorption routes can be found in Figure 1.

Current research aimed to describe the effect of the mentioned cellulose derivatives on the in situ gelling properties of gellan gum, their effect on the colloidal characteristics of the model, risperidone-loaded polymeric micelle formulation’s colloidal characteristics, and in vitro release and permeability profile. As a dual-level controlled release platform, it has not yet been widely studied, especially not for nasal drug delivery purposes. The added value lies in the dual barrier for the encapsulated drug, where there is an inner polymeric micelle encapsulating and protecting the drug, whilst the outer gellan gum–cellulose derivative polymeric matrix tailors the drug release and permeation profile. Generally, polymeric micelles are characterized by a burst-like drug release profile, and the hypothesis was that this extensive burst could be engineered into a sustained release profile. The basis for this hypothesis is that upon contact with the nasal mucosa, a sol–gel transition occurs, forming a thin hydrogel film on the surface.

## 2. Results and Discussion

### 2.1. Characterization of Gellan Gum-Based Hydrogels

#### 2.1.1. Determination of In Situ Gelling Properties of Hydrogels

At first, the freeze-dried formulations were dissolved individually in purified water, and in a second vial, in a simulated nasal electrolyte solution (SNES). SNES provided an adequate calcium ion concentration to initiate the gelation, and the samples were visually observed, along with comparing them between the ion-free and SNES dissolution media. In most cases, soft, transparent, and translucent gels were obtained, but at lower concentrations of gellan gum and each cellulose derivative, only slightly viscous liquids were observed. They still adhered to the vials, with a slow slipping motion overturning them, acting like classic mucilages. In purified water, no gelation occurred (Table 1). Typically, gellan gum forms firm gels over 0.5% concentrations; however, with the added cellulose derivatives, this value decreased, as the formed gels were able to adhere to the bottom of the vials instead of slipping.

#### 2.1.2. Clarity, pH, and Gel Content Determination

Visual observation was utilized to describe the formulation, where most of the formulations had transparent clarity, except for the ones with high concentrations of gellan gum and HEC, HPMC, and all formulations with MC. The pH of the formulations ranges between 5.2 and 5.8 on average, in correlation with the slightly acidic pH of the nasal cavity, allowing suitable application with a decreased chance of irritation or hypersensitivity due to pH shift [33]. The drug content was also measured with no significant differences, proving the proper encapsulation of the polymeric micelles inside the gel matrices (Table 2).

#### 2.1.3. Determination of Expansion Coefficient and Water-Holding Capacity

The expansion coefficient (S), also known as the swelling ratio, was measured to further characterize the formulations. There is a similar tendency in all blocks of formulations, meaning that with an increase in the concentration of gellan gum and the cellulose-derived polymer, the formulations swelled more. The higher expansion describes a more porous or loosely crosslinked network, which is based on the increased hydrophilicity per volume of the formulation. Highly swollen gels may also be weaker mechanically, which is expected for nasal formulations since highly firm hydrogels may cause discomfort in the nasal cavity. Porous hydrogels also allow the mucociliary clearance to flow through the gel matrices whilst providing higher residence time and decreased hindrance for diffusion from the matrix to outer space. The water-holding capacity is also of paramount importance since the encapsulated nanoparticles are highly solubilized and tend to diffuse with water, providing drug release. The higher the water-holding capacity, the more sustained the release profile expected. This tendency is like the expansion coefficient values, and the highest values can be found in the case of the highest gellan gum concentrations with HPMC (Table 3).

#### 2.1.4. Rheological Investigations

Similar to other polysaccharides, such as alginate and carrageenan, gellan gum shows gelation in the presence of cations. As the temperature decreases, gellan gum chains form a double helix, resulting in junction zones and the formation of a gel structure. The presence of the cation has a strong influence on this gelation process [34].

**Figure 2:**
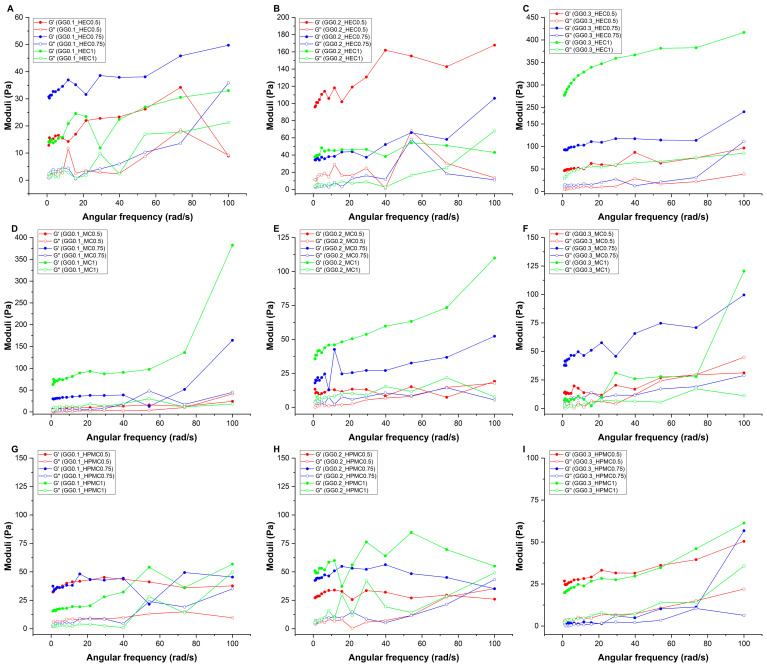
Elastic (G′) and viscous (G″) moduli curves as a function of the angular frequency in the case of gellan gum-based hydrogels, combined with (**A**–**C**) hydroxyethyl cellulose, (**D**–**F**) methylcellulose, and (**G**–**I**) hydroxypropyl-methylcellulose. Formulations were named via the following system: GG (gellan gum) with its respective concentration (% *w*/*v*), followed by the concentration of either HEC (hydroxyethyl cellulose), methylcellulose (MC), or hydroxypropyl-methylcellulose (HPMC), also in the unit of % *w*/*v*.

Nasal fluid contains divalent and monovalent cations as well. Compared with the monovalent cations, the divalent cations promote gelation much more strongly due to the chemical interaction between the ion and the carboxylate groups of the gluconic acid [35]. In our study, the formulations were mixed with SNES, and the final gel structures were analyzed with rheological tests. The mechanical strength of the gels was characterized by rheological investigations, and the obtained storage moduli (G′) and loss moduli (G″) were recorded against the angular frequency (Figure 2).

In most cases, G′ is higher than G″, proving the gel structure of them, but for GG0.1_MC0.2, this changed, and the G″ is higher than G′, reflecting a viscous liquid or still a sol state. In all cases, the moduli showed high-frequency dependency, which indicates a weaker gel structure. Generally, the higher the slope of the frequency sweep curve is, the weaker the gel structure [36].

In the case of the GG–HEC complex, higher GG concentrations increased the storage moduli and the strength of the gel, while the strongest interaction could be observed in the GG0.3_HEC1 formulations. This polymer blend can result in a semi-interpenetrating polymer network (semi-IPN), where the interpenetration of the two polymers can enhance the gel properties (in the case of the GG0.3_HEC1 sample, the highest G′ can be seen, the G′ modulus is an order of magnitude larger than the G” modulus, and this formulation demonstrated less frequency dependency). In the case of the combination of GG with MC or HPMC, there is no such change; neither the amount of GG nor the amount of added polymer results in an increase to the same extent as in the case of GG-HEC combinations. The GG-MC-containing samples presented a moderate increase in the modulus with the addition of the MC, while GG-HPMC samples presented a weakly similar gel structure. This behavior can likely be explained by the observation that the MC and HPMC can form a weaker gel structure in the presence of ions, while the more hydrophilic HEC can strengthen the gel structure, forming a semi-IPN with the GG.

Based on the modulus data and their high-frequency dependency, the formulations show a weak elastic gel behavior, dependent on the concentration of added gellan gum or cellulose derivatives. In conclusion, in rheological terms, the combination of GG and HEC can be beneficial.

### 2.2. Characterization of Polymeric Micelles

Generally, the addition of other polymeric components to a polymeric micelle solution can influence the nanoparticle characteristics, including the micelle size, size distribution, and the degree of solubilization, as well as the encapsulation efficiency. The results from dynamic light-scattering measurements and the encapsulation efficiency determination can be found in Table 4. The micelle size, expressed as the average hydrodynamic diameter (D_H_), slightly increased compared to the gel-free polymeric micelle solution (RIS-PM), but there was no tendency amongst the values. The average polymeric micelle size lies between 10 and 200 nm, and all values can be found in that range, meaning that the particles remained colloidally stable upon gelation. The same can be claimed about the micelle size distribution, which is expressed as the polydispersity index. All values increased slightly but remained under 0.300, which means they have a monodisperse size distribution, providing the feasibility of uniform drug release and permeation profile. The encapsulation efficiency slightly decreased in a manner corresponding to the increase in size and size distribution, connoting a small drug leakage from the micellar core to the gel matrices. However, the differences are not significant; the values are still considerably high, providing excellent solubilization capacity.

### 2.3. Nasal Applicability Studies

#### 2.3.1. In Vitro Mucoadhesion Study

The mucoadhesive nature of the polymeric micelle solution (RIS-PM) was first investigated, which showed a low value, only capable of weak adhesion to the nasal mucosa. On the other hand, all formulations have an increased mucoadhesive profile, with a concentration-dependent tendency. With the increase in the gellan gum concentration, the mucoadhesiveness increased. Gellan gum polymers can intertwine with mucin glycoproteins found in the nasal mucosa; thus, this physical entanglement also prolongs residence time. Polar hydroxyl groups can also take part in hydrogen bonding and electrostatic interactions due to the anionic nature of gellan gum [37]. The applied cellulose derivatives can also hydrate and swell up in the nasal mucosa, forming gel-like structures and a thin film layer on the surface. Hydrogen bonding also plays a crucial part in the mucoadhesion process, further enhancing the synergy between gellan gum and the cellulose derivatives (Table 5).

#### 2.3.2. In Vitro Drug Release Study

**Figure 3:**
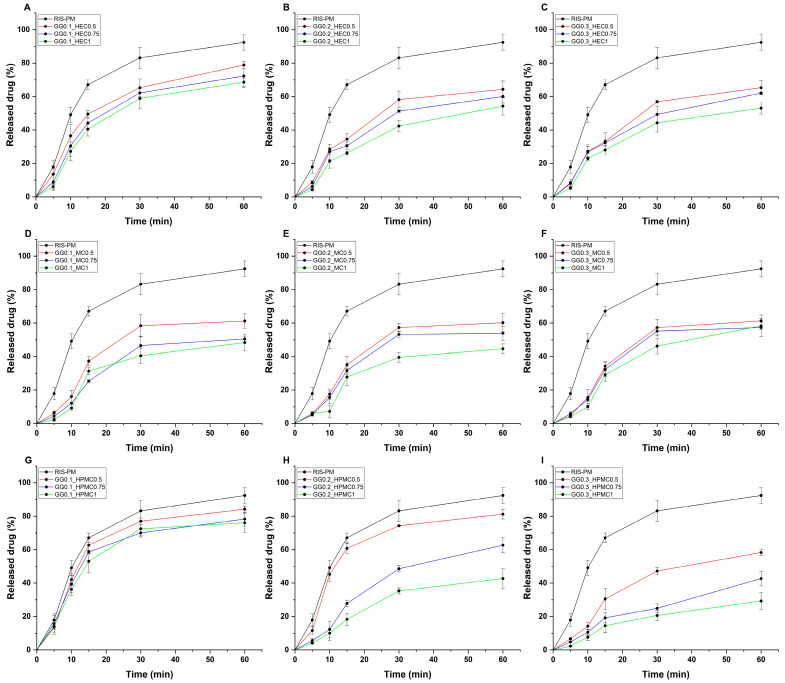
In vitro drug release profiles of gellan gum-based hydrogels, combined with (**A**–**C**) hydroxyethyl cellulose, (**D**–**F**) methylcellulose, and (**G**–**I**) hydroxypropyl-methylcellulose, compared with gel matrix-free polymeric micelles (RIS-PM). Experiments were conducted at simulated nasal conditions. Data are expressed as average ± SD (n = 3). Formulations were named via the following system: GG (gellan gum) with its respective concentration (% *w*/*v*), followed by the concentration of either HEC (hydroxyethyl cellulose), methylcellulose (MC), or hydroxypropyl-methylcellulose (HPMC), also in the unit of % *w*/*v*. RIS-PM represents the polymeric micelle formulation dispersed in solely purified water.

The in vitro drug release studies are corroborated by the results from the rheological investigations. The limitation of this study is the setup, as in the case of a rheological measurement, the hydrogels are as flat as possible, similar to the conditions on the nasal mucosa, whilst in the case of the drug release study, they form a block after gelation in the dialysis bag. The higher the strength of the gel, the more sustained the drug release profile obtained. In comparison with RIS-PM, the smallest difference can be found in the case of the GG0.1_HPMC0.5/0.75/1 formulations, whilst the highest difference is with the GG0.3_HPMC1 formulation. Generally, the increase in the cellulose derivatives prolonged the drug release in all cases (Figure 3). Polymeric micelles are described with a diffusion-controlled release due to their high water solubility, which is hindered by gellan gum. Gellan gum forms tight three-dimensional networks and physically traps drug molecules, making it difficult for them to diffuse quickly. The increase in viscosity also traps the micelles; thus, their rapid-release tendency is also hindered, as they are not prone to dilution in aqueous matrices. Mobility is also a key element, as the polymer entanglement also restricts micelle mobility due to electrostatic interactions or hydrogen bonding amongst the gellan gum–cellulose derivative matrix and the hydrophilic corona of the micelles. The higher the water-holding capacity, the more sustained the profile observed. Also, the higher gellan gum content results in denser gels, responsible for slower release. Upon dilution and under constant flow, the gellan gum matrices slowly erode, releasing the drug over time.

#### 2.3.3. In Vitro Drug Permeation Study

The permeability study was conducted to determine the passive diffusion tendencies of the formulations, and the flux (J) and permeability coefficient (Kp) were calculated for the 1 h measurement period. The flux values correspond to the drug release kinetics, as the sustained release profiled formulations also have a decreased flux value. Theoretically, this means that the overall bioavailability of the formulations is worse compared to the initial RIS-PM; however, the highly soluble polymeric micelles are washed away easily from the mucosal surface. The increased mucoadhesion of the in situ gels aids in the possibility of higher permeability through the mucosal surface, as it would not be washed away. Concentration dependence amongst the formulations can be seen in Table 6, as with the increase in gellan gum or the respective cellulose derivatives’ concentration, the permeation slowed down over the period.

## 3. Conclusions

This descriptive-based research presents thorough information about gellan gum interactions with various cellulose derivatives during nasal administration. The research examined the combined physical properties and cooperative functions of these polymers during formulation development, which benefits the creation of advanced drug delivery systems. As an anionic polysaccharide, gellan gum shows promise for mucoadhesive drug delivery because it creates ion-sensitive gels when it meets nasal mucosa surfaces. The gel systems containing gellan gum with hydroxypropyl methylcellulose (HPMC), hydroxyethylcellulose (HEC), and methylcellulose (MC) displayed enhanced structural integrity and drug release control capabilities.

This study revealed how added cellulose derivatives reacted with concentration changes in their mixtures. The nasal environment simulations revealed that higher polymer concentrations led to the formation of more robust gels. The enhanced gel strength extends the time the formulation stays within the nasal cavity, thus boosting the bioavailability of route-specific medications. The rheological measurements revealed that the solution transformed from a fluid state to a semi-solid gel state, thus enabling extended drug retention and absorption at the absorption site. This is especially true in the case of a high GG concentration of 0.3% *w*/*v* for all cellulose derivatives, where the retained amount of drug accumulated in a sustained drug release and permeation profile. This is also corroborated by the high water-holding capacity values of over 80%.

This research examined the common drug release problem of polymeric micelles, which causes immediate drug release. Polymeric micelles demonstrate excellent capabilities for dissolving poorly soluble drugs while protecting them from breakdown, but their fast release at the start reduces their therapeutic effect. The combination of gellan gum with cellulose derivatives resulted in hydrogel matrices that showed excellent potential to resolve the rapid drug release problem. The combined hydrogel materials functioned as permeation barriers, which reduced drug diffusion speed from the system, thus controlling its release profile. The result is a more controlled and sustained drug release pattern, which is highly desirable in nasal drug delivery to ensure consistent therapeutic levels and minimize the dosing frequency.

This research confirmed that combining gellan gum with cellulose derivatives shows promise for nasal administration, while providing detailed information about concentration-based formulation optimization. The adjustable properties of gel strength and drug release profiles through polymer composition modifications establish hydrogel systems as flexible solutions for current nasal drug delivery technology problems.

## 4. Materials and Methods

### 4.1. Materials

Pluronic^®^ F-108 (P108) (PEG_136_-PPG_52_-PEG_136_ (poly(ethylene-glycol)–block–poly(propylene-glycol)–block–poly(ethylene glycol); average molecular weight: 14,600 Da), Pluronic^®^ F-127 (P127) (PEG_95_-PPG_62_-PEG_95_ (poly(ethylene-glycol)–block–poly(propylene-glycol)–block–poly(ethylene glycol); average molecular weight: 12,500 Da), and risperidone (RIS; 3-[2-[4-(6-fluoro-1,2-benzisoxazol-3-yl)-1-piperidinyl]ethyl]-6,7,8,9-tetrahydro-2-methyl-4H-pyrido[1,2-a]pyrimidin-4-one; water solubility: <0.1 mg/mL; logP: 2.7; molecular weight: 410.49 g/mol; oral bioavailability: 70%) were acquired from Sigma-Aldrich Co., Ltd. (Budapest, Hungary). Gellan gum (GG, Phytagel^®^), hydroxyethyl cellulose (HEC), methylcellulose (MC), and hydroxypropyl-methylcellulose were utilized as gel-forming agents, also acquired from Sigma-Aldrich Co., Ltd. For the in vitro nasal investigations, simulated nasal electrolyte solution (SNES) was utilized with the following composition: 2.98 g/L of potassium chloride, 8.77 g/L of sodium chloride, and 0.59 g/L of anhydrous calcium chloride in 1000 mL of purified water, adjusted to a pH of 5.6.

### 4.2. Quantitative Analysis of Risperidone via High-Performance Liquid Chromatography

To quantify risperidone during the measurements, high-performance liquid chromatography was used by an Agilent Infinity Instrument (Agilent Technologies, Santa Clara, CA, USA). The stationary phase was a Kinetex^®^ C18 column (5 µm, 150 mm × 4.6 mm (Phenomenex, Torrance, CA, USA)). The mobile phases were the following: acidic purified water (0.1% *w*/*v* formic acid) (A) and acidic acetonitrile (0.1% *w*/*v* formic acid) (B). The injection volume was 10 µL with a set temperature of 25 °C. Isocratic elution was performed for 6 min. Chromatograms were detected at 280 ± 4 nm using a UV-Vis diode array detector. The retention time was 2.65 min. The chromatograms were evaluated using ChemStation B.04.03. Software (Agilent Technologies, Santa Clara, CA, USA). The limit of detection (LOD) and limit of quantification (LOQ) were 4.78 and 15.75 ppm, respectively. Calibration was performed between 5 and 100 µg/mL, with a determined coefficient of linearity (R^2^) of 0.9999.

### 4.3. Formulation of Risperidone-Loaded Polymeric Micelles

For the formulation of risperidone-loaded polymeric micelles, the direct freeze-drying method was used, with a formulation based on prior results [38]. First, 5 mg of risperidone was dissolved in 10 mL of tert-butyl alcohol and mixed with the same volume of the aqueous solution of 80 mg of P-127 and 20 mg of P-108. Samples were placed in dials and freeze-dried using a ScanVac CoolSafe 100-9 laboratory apparatus (LaboGene, ApS, Lynge, Denmark). First, samples were kept freezing at –40 °C for 12 h under 0.013 mbar pressure, followed by 6 h of secondary drying at 25 °C at 0.013 mbar. Freeze-dried cakes were dissolved in 1 mL of purified water or gel bases for further investigations.

### 4.4. Study Design of Gellan Gum-Based Soft Hydrogels

Gellan gum was dissolved in 80 °C purified water in the respective concentrations, whilst hydroxyethyl cellulose (HEC), methylcellulose (MC), and hydroxypropyl-methylcellulose (HPMC) were dissolved at an ambient temperature. Equal parts of the gellan gum solution were mixed with the cellulose derivative solutions. Altogether, 27 formulations were tested, with the final concentration of each polymer described in Table 7.

### 4.5. Characterization of Gellan Gum-Based Soft Hydrogels

#### 4.5.1. Determination of In Situ Gelling Properteis of Hydrogels

To determine the in situ gelling tendencies of the formulations, freeze-dried samples were dissolved in the simulated nasal electrolyte solution. Samples were incubated at 32 ± 1 °C. Visible gelling time was measured, and the gel formation was described based on liquidity. All measurements were carried out in triplicate (n = 3), and the results are expressed as the average ± SD.

#### 4.5.2. Clarity, pH, and Drug Content Study

The clarity of the formulations was determined posterior to gelation by visual examination against white and black backgrounds under light. The pH of the formulations was measured by dipping a pH meter (WTW^®^ inoLab^®^ pH 7110 laboratory pH tester, Thermo Fisher Scientific, Budapest, Hungary). For the drug content determination, 1 mL of the gel was destroyed and diluted to 10 mL with methanol. The dispersions were vortexed for 10 min, followed by filtration through a 0.45 µm pore-sized polyether sulfone (PES) membrane. The RIS concentration was determined in the filtrate by HPLC. All measurements were carried out in triplicate (n = 3), and the results are expressed as the average ± SD.

#### 4.5.3. Determination of Expansion Coefficient

The expansion coefficient was measured according to a previously described methodology [5,39]. First, 1–1 mL of the formulation was mixed with 0.5 mL SNES (pH 5.6) in a graduated test tube; the total liquid volume (V_1_) was noted (1.50 mL). The volume of the gel after gelation (V_g_) was calculated by the addition of 2.0 mL of SNES, where the obtained volume was considered the total volume (V_t_). The following equations were used to calculate the expansion coefficient value (S):

(1) $$V_{g}\left(\mathrm{mL}\right)=V_{t}-2$$

(2) $$S\left(\%\right)=\frac{\left(V_{g}-V_{1}\right)}{V_{1}}\times 100$$

All measurements were carried out in triplicate (n = 3), and the results are expressed as the average ± SD.

#### 4.5.4. Determination of Water-Holding Capacity

Similarly to the expansion coefficient investigation, 1–1 mL of the formulation was measured, and 0.5 mL of SNES was added to them in a pre-weighed centrifuge tube. The weight of the gel was noted as W_1_. Samples were centrifuged at 8000× *g* (10 min, 4 °C), then the supernatant was removed from the gel, and the remaining mass was weighed (W_2_). The water-holding capacity (WHC) was measured via the following equation:

(3) $$WHC\;(\%)=\frac{W_{2}}{W_{1}}\times 100$$

All measurements were carried out in triplicate (n = 3), and the results are expressed as the average ± SD.

#### 4.5.5. Determination of Gel Strength

The rheological investigations were performed with a Physica MCR302 Rheometer (Anton Paar, Graz, Austria). The measuring device was a cone and plate type (cone angle was 1°) with a diameter of 25 mm. The applied gap height during the tests was 0.05 mm. An amplitude sweep test was performed using a 10 rad/s angular frequency. The storage (G′) and loss (G″) moduli of each gel were determined by increasing the shear strain (γ) from 0.1 to 100%. The linear viscoelastic region (LVER) (yield point) and the crossover point of G′ and G″ (flow point) were determined. The frequency sweep tests were performed at 0.1% shear strain within the angular frequency range of 1–100 rad/s. For comparison and analysis, the storage (G′) modulus and the loss factors (tanδ) were expressed as 1 rad/s. All measurements were carried out in triplicate (n = 3), and the results are expressed as average ± SD.

### 4.6. Characterization of Polymeric Micelles

#### 4.6.1. Dynamic Light-Scattering Measurements

Dynamic light-scattering measurements were carried out to determine micelle size (expressed as the average hydrodynamic diameter (D_H_)) and micelle size distribution (expressed as the polydispersity index (PdI)) using the Malvern Nano ZS Zetasizer (Malvern Instruments, Worcestershire, UK). Samples were placed in folded capillary cells. The measurements were carried out with a refractive index of 1.335 and a temperature of 32 °C. All measurements were carried out in triplicate (n = 3), and the results are expressed as average ± SD.

#### 4.6.2. Determination of Encapsulation Efficiency

For the determination of the encapsulation efficiency (EE), 1 mL of samples was placed in Spin-X^®^ centrifuge tubes (Costar, Salt Lake City, UT, USA), which contain a cellulose acetate membrane filter with a 0.22 µm cut-off pore diameter. Samples were filtered through this membrane via centrifugation, using a Hermle Z323 K high-performance refrigerated centrifuge (Hermle AG, Gosheim, Germany). Filtration was performed at 13,500 rpm at 4 °C for 30 min, followed by quantitative measurements via HPLC. All measurements were carried out in triplicate (n = 3), and the results are expressed as average ± SD. EE was calculated via the following equation:

(4) $$EE\;(\%)=\frac{measured\;RIS\;(\mathrm{mg})}{initial\;RIS\;(\mathrm{mg})}\times 100$$

### 4.7. Nasal Applicability Studies

#### 4.7.1. In Vitro Mucoadhesion Study

Tensile tests were performed to evaluate the mucoadhesive nature of the formulations, using a TA-XT Plus Texture Analyser (Metron Ltd., Budapest, Hungary) equipped with a 5 kg load cell and a 1 cm diameter cylinder probe. Formulations were dispersed in SNES and brought into contact with a 25 mm diameter filter-paper disc, which was moistened with 50 µL of 8% *w*/*w* porcine mucin (type III) dispersion. Then, 20 µL samples were applied to the filter paper, which was secured to the cylinder probe, and placed into contact with the mucin dispersion. For 3 min, a 2500 mN preload was applied, after which the cylinder probe was moved upward at a set speed of 2.5 mm/min to separate the contact surfaces. The mucoadhesivity was assessed based on the adhesive force (F, mN) and the adhesive work (A, mN × mm). All measurements were carried out in triplicate (n = 4), and the results are expressed as the average ± SD.

#### 4.7.2. In Vitro Drug Release Study

For the drug release study, formulations were placed in dialysis tubes (Spectra/Por^®^ Dialysis Membrane with a 12–14 kDa MWCO (Spectrum Laboratories Inch, Rancho Dominguez, CA, USA)) and immersed in 100 mL of SNES under sink conditions. Measurements were performed under 100 rpm of paddle rotation at 32 °C. Aliquots were taken at predetermined time points up to 60 min, followed by quantification via HPLC. All measurements were carried out in triplicate (n = 3), and the results are expressed as the average ± SD.

#### 4.7.3. In Vitro Drug Permeation Study

A modified Side-bi-Side^®^ type horizontal diffusion cell was utilized for the in vitro drug permeation study, where the donor and acceptor compartments were separated with a cellulose membrane impregnated with isopropyl myristate. The donor compartment was 9.0 mL of each formulation dissolved in SNES, while the acceptor compartment was 9.0 mL of pH 7.4 phosphate buffer solution (PBS). The diffusion surface was 0.785 cm^2^. Aliquots were taken at predetermined points up to 60 min, followed by quantification via HPLC. All measurements were carried out in triplicate (n = 3), and the results are expressed as average ± SD.

Flux (J) was measured via Equation (5), where m_t_ is the permeated drug amount at *t* time and *A* is the surface of the membrane.

(5) $$J=\frac{m_{t}}{t\times A}$$

The permeability coefficient (K_p_) was determined from the flux and the drug concentration in the donor phase (Cd).

(6) $$K_{p}=\frac{J}{C_{d}}$$

## Figures and Tables

**Table 1:** Gelation of gellan gum–cellulose derivative composites based on their composition in simulated nasal electrolyte solution. “+” means visible gelation at 32 °C, whilst still capable of slipping from the vials. “++” shows a transition between slipping hydrogels and firm hydrogels, and “+++” shows firm hydrogel formation without slipping.

	Gellan Gum Concentration (% *w*/*v*)		
	0.1	0.2	0.3
HEC 0.5% *w*/*v*	+	+	+
HEC 0.75% *w*/*v*	+	++	++
HEC 1% *w*/*v*	++	+++	+++
MC 0.5% *w*/*v*	+	+	+
MC 0.75% *w*/*v*	+	++	++
MC 1% *w*/*v*	++	++	+++
HPMC 0.5% *w*/*v*	+	++	++
HPMC 0.75% *w*/*v*	++	+++	+++
HPMC 1% *w*/*v*	+++	+++	+++

**Table 2:** Clarity, pH, and drug content of the various hydrogel formulations. Data are expressed as average ± SD (n = 3). Formulations were named via the following system: GG (gellan gum) with its respective concentration (% *w*/*v*), followed by the concentration of either HEC (hydroxyethyl cellulose), methylcellulose (MC), or hydroxypropyl-methylcellulose (HPMC), also in the unit of % *w*/*v*.

Formulation	Clarity	pH	Drug Content (%)
GG0.1_HEC0.5	transparent	5.47 ± 0.21	98.76 ± 1.42
GG0.1_HEC0.75	transparent	5.63 ± 0.14	95.84 ± 0.67
GG0.1_HEC1	transparent	5.52 ± 0.2	100.15 ± 1.83
GG0.2_HEC0.5	transparent	5.71 ± 0.34	99.27 ± 0.91
GG0.2_HEC0.75	transparent	5.49 ± 0.11	96.43 ± 1.15
GG0.2_HEC1	transparent	5.56 ± 0.26	97.12 ± 0.44
GG0.3_HEC0.5	transparent	5.34 ± 0.19	100.98 ± 1.76
GG0.3_HEC0.75	cloudy	5.71 ± 0.35	96.88 ± 0.58
GG0.3_HEC1	cloudy	5.39 ± 0.24	99.95 ± 1.27
GG0.1_MC0.5	cloudy	5.44 ± 0.15	97.59 ± 0.73
GG0.1_MC0.75	cloudy	5.34 ± 0.09	98.33 ± 1.94
GG0.1_MC1	cloudy	5.56 ± 0.18	100.41 ± 0.39
GG0.2_MC0.5	cloudy	5.48 ± 0.31	95.67 ± 1.02
GG0.2_MC0.75	cloudy	5.59 ± 0.14	99.78 ± 1.68
GG0.2_MC1	cloudy	5.50 ± 0.26	101.12 ± 0.87
GG0.3_MC0.5	cloudy	5.31 ± 0.12	98.03 ± 0.6
GG0.3_MC0.75	cloudy	5.52 ± 0.27	97.45 ± 1.33
GG0.3_MC1	cloudy	5.24 ±0.18	96.17 ± 1.89
GG0.1_HPMC0.5	transparent	5.67 ± 0.33	100.64 ± 0.49
GG0.1_HPMC0.75	transparent	5.43 ± 0.21	95.26 ± 1.11
GG0.1_HPMC1	transparent	5.38 ± 0.08	99.10 ± 0.85
GG0.2_HPMC0.5	transparent	5.59 ± 0.29	98.54 ± 1.59
GG0.2_HPMC0.75	transparent	5.25 ± 0.31	101.3 ± 0.95
GG0.2_HPMC1	transparent	5.73 ± 0.15	95.93 ± 1.21
GG0.3_HPMC0.5	transparent	5.61 ± 0.24	97.88 ± 0.36
GG0.3_HPMC0.75	transparent	5.46 ± 0.34	99.51 ± 1.5
GG0.3_HPMC1	cloudy	5.34 ± 0.11	96.75 ± 0.78

**Table 3:** Expansion coefficient (S) and water-holding capacity (WHC) of the various gel formulations. Data are expressed as average ± SD (n = 3). Formulations were named via the following system: GG (gellan gum) with its respective concentration (% *w*/*v*), followed by the concentration of either HEC (hydroxyethyl cellulose), methylcellulose (MC), or hydroxypropyl-methylcellulose (HPMC), also in the unit of % *w*/*v*. * describes the expansion coefficient as high (>5%) with at least 80% water-holding capacity, suitable for longer-term hydrogel stability.

Formulation	S (%)	WHC (%)
GG0.1_HEC0.5	1.25 ± 0.09	75.07 ± 3.14
GG0.1_HEC0.75	1.62 ± 0.42	77.14 ± 1.72
GG0.1_HEC1	4.18 ± 0.37	77.65 ± 4.05
GG0.2_HEC0.5	4.77 ± 0.33	78.09 ± 2.89
GG0.2_HEC0.75	4.43 ± 0.18	78.86 ± 0.76
GG0.2_HEC1	5.01 ± 0.21	79.54 ± 1.38
* GG0.3_HEC0.5	7.05 ± 0.28	86.44 ± 4.62
* GG0.3_HEC0.75	7.31 ± 0.07	87.21 ± 2.47
* GG0.3_HEC1	7.62 ± 0.41	88.03 ± 0.59
GG0.1_MC0.5	2.03 ± 0.12	75.31 ± 3.91
GG0.1_MC0.75	2.47 ± 0.35	75.61 ± 4.27
GG0.1_MC1	3.31 ± 0.25	80.27 ± 2.05
* GG0.2_MC0.5	5.29 ± 0.44	81.43 ± 1.94
* GG0.2_MC0.75	5.52 ± 0.16	82.15 ± 0.84
* GG0.2_MC1	5.88 ± 0.39	82.98 ± 3.53
* GG0.3_MC0.5	7.88 ± 0.14	88.56 ± 4.11
* GG0.3_MC0.75	8.17 ± 0.31	89.72 ± 2.71
* GG0.3_MC1	8.39 ± 0.24	90.35 ± 1.15
GG0.1_HPMC0.5	2.88 ± 0.29	75.96 ± 4.77
GG0.1_HPMC0.75	3.62 ± 0.18	76.28 ±0.91
GG0.1_HPMC1	3.95 ± 0.43	83.74 ± 3.31
GG0.2_HPMC0.5	6.11 ± 0.22	76.73 ± 2.24
GG0.2_HPMC0.75	6.49 ± 0.11	84.36 ± 1.59
GG0.2_HPMC1	6.78 ± 0.34	85.02 ± 3.76
* GG0.3_HPMC0.5	8.64 ± 0.27	85.67 ± 2.63
* GG0.3_HPMC0.75	8.93 ± 0.08	90.97 ± 0.68
* GG0.3_HPMC1	9.14 ± 0.32	91.84 ± 4.39

**Table 4:** Effect of the various in situ gel compositions on the characteristics of polymeric micelles: micelle size (DH), polydispersity index (PdI), and encapsulation efficiency (EE). Data are expressed as average ± SD (n = 3). Formulations were named via the following system: GG (gellan gum) with its respective concentration (% *w*/*v*), followed by the concentration of either HEC (hydroxyethyl cellulose), methylcellulose (MC), or hydroxypropyl-methylcellulose (HPMC), also in the unit of % *w*/*v*. RIS-PM represents the polymeric micelle formulation dispersed in solely purified water.

Formulation	D_H_ (nm)	PdI	EE (%)
RIS-PM	30.17 ± 2.6	0.115 ± 0.012	84.15 ± 2.85
GG0.1_HEC0.5	39.41 ± 4.32	0.183 ± 0.014	81.42 ± 3.72
GG0.1_HEC0.75	45.19 ± 1.87	0.155 ± 0.022	79.11 ± 1.84
GG0.1_HEC1	39.43 ± 3.45	0.260 ± 0.006	83.24 ± 5.60
GG0.2_HEC0.5	37.12 ± 0.91	0.110 ± 0.031	78.97 ± 4.15
GG0.2_HEC0.75	35.01 ± 2.78	0.246 ± 0.017	76.55 ± 2.96
GG0.2_HEC1	40.14 ± 1.24	0.225 ± 0.008	81.03 ± 6.03
GG0.3_HEC0.5	41.28 ± 4.61	0.125 ± 0.025	80.15 ± 0.89
GG0.3_HEC0.75	40.69 ± 3.08	0.211 ± 0.019	80.83 ± 5.12
GG0.3_HEC1	41.75 ± 0.73	0.243 ± 0.012	76.12 ± 2.41
GG0.1_MC0.5	36.25 ± 2.15	0.194 ± 0.029	78.95 ± 1.27
GG0.1_MC0.75	35.66 ± 4.04	0.232 ± 0.007	78.52 ± 4.88
GG0.1_MC1	39.09 ± 1.39	0.145 ± 0.034	79.45 ± 6.45
GG0.2_MC0.5	36.79 ± 2.52	0.120 ± 0.011	83.68 ± 0.66
GG0.2_MC0.75	37.32 ± 3.91	0.158 ± 0.007	83.14 ± 3.09
GG0.2_MC1	42.10 ± 4.76	0.173 ± 0.026	84.25 ± 2.18
GG0.3_MC0.5	42.84 ± 0.68	0.189 ± 0.015	83.12 ± 1.96
GG0.3_MC0.75	43.29 ± 1.03	0.268 ± 0.032	82.63 ± 5.81
GG0.3_MC1	43.88 ± 2.96	0.114 ± 0.009	79.02 ± 4.53
GG0.1_HPMC0.5	41.05 ± 0.59	0.201 ± 0.017	85.64 ± 0.58
GG0.1_HPMC0.75	37.88 ± 3.66	0.214 ± 0.025	85.01 ± 6.28
GG0.1_HPMC1	38.41 ± 1.75	0.267 ± 0.018	86.77 ± 3.43
GG0.2_HPMC0.5	37.42 ± 4.29	0.132 ± 0.003	83.67 ± 2.79
GG0.2_HPMC0.75	36.11 ± 2.21	0.164 ± 0.027	79.02 ± 1.09
GG0.2_HPMC1	39.05 ± 3.18	0.245 ± 0.035	81.44 ± 6.67
GG0.3_HPMC0.5	39.73 ± 0.84	0.223 ± 0.016	78.60 ± 0.94
GG0.3_HPMC0.75	44.37 ± 4.45	0.226 ± 0.013	78.53 ± 4.34
GG0.3_HPMC1	44.96 ± 2.39	0.198 ± 0.030	79.33 ± 3.86

**Table 5:** Mucoadhesive force and work of the in situ gelling formulations compared with the raw polymeric micelle solution. Data are expressed as average ± SD (n = 3). Formulations were named via the following system: GG (gellan gum) with its respective concentration (% *w*/*v*), followed by the concentration of either HEC (hydroxyethyl cellulose), methylcellulose (MC), or hydroxypropyl-methylcellulose (HPMC), also in the unit of % *w*/*v*. RIS-PM represents the polymeric micelle formulation dispersed in solely purified water.

Formulation	Mucoadhesive Force (mN)	Mucoadhesive Work (mN × mm)
RIS-PM	765.98 ± 102.59	36.11 ± 3.37
GG0.1_HEC0.5	1394.17 ± 211.58	66.56 ± 5.12
GG0.1_HEC0.75	1662.03 ± 120.37	74.71 ± 6.95
GG0.1_HEC1	1881.46 ± 208.50	81.44 ± 11.71
GG0.2_HEC0.5	1924.89 ± 315.07	85.28 ± 6.21
GG0.2_HEC0.75	1975.33 ± 266.71	99.87 ± 7.63
GG0.2_HEC1	2308.67 ± 145.98	101.81 ± 22.62
GG0.3_HEC0.5	1854.01 ± 164.62	86.17 ± 5.19
GG0.3_HEC0.75	2005.74 ± 134.97	92.34 ± 9.78
GG0.3_HEC1	2446.29 ± 137.19	103.23 ± 12.28
GG0.1_MC0.5	1269.91 ± 157.18	39.75 ± 4.61
GG0.1_MC0.75	1575.59 ± 238.04	57.12 ± 8.43
GG0.1_MC1	1872.55 ± 326.27	75.89 ± 6.88
GG0.2_MC0.5	1937.25 ± 194.72	83.45 ± 11.85
GG0.2_MC0.75	2230.86 ± 270.36	91.03 ± 10.43
GG0.2_MC1	2513.87 ± 311.94	111.58 ± 8.71
GG0.3_MC0.5	2130.93 ± 354.26	87.85 ± 11.89
GG0.3_MC0.75	2312.87 ± 147.63	93.69 ± 6.52
GG0.3_MC1	2716.81 ± 121.98	124.01 ± 9.67
GG0.1_HPMC0.5	1405.28 ± 376.45	58.29 ± 8.72
GG0.1_HPMC0.75	1559.84 ± 244.01	75.96 ± 4.63
GG0.1_HPMC1	1792.37 ± 175.72	85.53 ± 6.95
GG0.2_HPMC0.5	1646.12 ± 338.89	62.41 ± 5.38
GG0.2_HPMC0.75	2004.28 ± 135.54	93.67 ± 7.14
GG0.2_HPMC1	2285.15 ± 282.33	127.35 ± 9.11
GG0.3_HPMC0.5	2466.61 ± 362.10	104.19 ± 3.89
GG0.3_HPMC0.75	2648.04 ± 191.46	115.82 ± 10.22
GG0.3_HPMC1	2775.43 ± 227.07	132.47 ± 6.74

**Table 6:** Flux (J) and permeability coefficient (K_p_) of the investigated formulations in comparison with in situ gelling agent-free risperidone-loaded polymeric micelles (RIS-PM). Data are expressed as average ± SD (n = 3). Formulations were named via the following system: GG (gellan gum) with its respective concentration (% *w*/*v*), followed by the concentration of either HEC (hydroxyethyl cellulose), methylcellulose (MC), or hydroxypropyl-methylcellulose (HPMC), also in the unit of % *w*/*v*. RIS-PM represents the polymeric micelle formulation dispersed in solely purified water.

Formulation	J (µg/cm^2^/h)	K_p_ (cm/h)
RIS-PM	317.51 ± 15.86	0.5715 ± 0.0142
GG0.1_HEC0.5	264.23 ± 13.26	0.4756 ± 0.0218
GG0.1_HEC0.75	251.27 ± 11.59	0.4523 ± 0.0293
GG0.1_HEC1	223.17 ± 10.84	0.4017 ± 0.0257
GG0.2_HEC0.5	198.12 ± 9.95	0.3566 ± 0.0159
GG0.2_HEC0.75	193.54 ± 15.28	0.3484 ± 0.0308
GG0.2_HEC1	188.04 ± 10.77	0.3385 ± 0.0225
GG0.3_HEC0.5	165.28 ± 12.06	0.2975 ± 0.0319
GG0.3_HEC0.75	148.89 ± 18.05	0.2680 ± 0.0123
GG0.3_HEC1	147.64 ± 12.39	0.2658 ± 0.0276
GG0.1_MC0.5	166.07 ± 18.09	0.2989 ± 0.0181
GG0.1_MC0.75	171.65 ± 13.68	0.3090 ± 0.0204
GG0.1_MC1	157.38 ± 11.19	0.2833 ± 0.0173
GG0.2_MC0.5	172.10 ± 16.04	0.3098 ± 0.0322
GG0.2_MC0.75	160.55 ± 18.62	0.2890 ± 0.0136
GG0.2_MC1	140.32 ± 11.34	0.2526 ± 0.0195
GG0.3_MC0.5	151.31 ± 13.70	0.2724 ± 0.0249
GG0.3_MC0.75	147.18 ± 20.38	0.2649 ± 0.0167
GG0.3_MC1	153.97 ± 16.00	0.2771 ± 0.0260
GG0.1_HPMC0.5	283.01 ± 10.81	0.5094 ± 0.0285
GG0.1_HPMC0.75	289.30 ± 16.53	0.5207 ± 0.0115
GG0.1_HPMC1	264.12 ± 18.06	0.4754 ± 0.0411
GG0.2_HPMC0.5	200.39 ± 12.67	0.3607 ± 0.0297
GG0.2_HPMC0.75	147.68 ± 17.61	0.2658 ± 0.0432
GG0.2_HPMC1	116.22 ± 20.29	0.2092 ± 0.0239
GG0.3_HPMC0.5	126.30 ± 19.56	0.2273 ± 0.0187
GG0.3_HPMC0.75	101.54 ± 11.12	0.1828 ± 0.0129
GG0.3_HPMC1	97.17 ± 15.99	0.1749 ± 0.0301

**Table 7:** Study design of gellan gum—HEC/MC/HPMC combined in situ gel formulations.

Gellan Gum Concentration (% *w*/*v*)	HEC/MC/HPMC Concentration (% *w*/*v*)
0.1	0.5
	0.75
	1.0
0.2	0.5
	0.75
	1.0
0.3	0.5
	0.75
	1.0

## Data Availability

Data can be acquired from the corresponding authors upon request.

## Abbreviations

The following abbreviations are used in this manuscript:

D_H_	average hydrodynamic diameter
EE	encapsulation efficiency
G′	storage moduli
G″	loss moduli
GG	gellan gum
HEC	hydroxyethyl cellulose
HPLC	High-performance liquid chromatography
HPMC	hydroxypropyl-methylcellulose
J	flux
K_p_	permeability coefficient
LOD	limit of detection
LOQ	limit of quantification
MC	methylcellulose
MWCO	molecular weight cut-off
P108	Pluronic F-108
P127	Pluronic F-127
PBS	phosphate buffered solution
PdI	polydispersity index
PEG	poly(ethylene glycol)
RIS	risperidone
S	expansion coefficient
SNES	simulated nasal electrolyte solution
WHC	water-holding capacity
